# Increasing the low lipid phosphate phosphatase 1 activity in breast cancer cells decreases transcription by AP-1 and expressions of matrix metalloproteinases and cyclin D1/D3

**DOI:** 10.7150/thno.37094

**Published:** 2019-08-14

**Authors:** Xiaoyun Tang, Todd P.W. McMullen, David N. Brindley

**Affiliations:** 1 Department of Biochemistry, University of Alberta, Edmonton, T6G 2S2, AB, Canada; 2 Cancer Research Institute of Northern Alberta, Edmonton, T6G 2S2, AB, Canada; 3 Department of Surgery, University of Alberta, Edmonton, Alberta, T6G 2R7, Canada

**Keywords:** Extracellular matrix, cJUN, cFOS, metastasis, tumor growth

## Abstract

Metastasis is the leading cause of mortality in breast cancer patients and lysophosphatidate (LPA) signaling promotes this process. LPA signaling is attenuated by lipid phosphate phosphatase-1 (LPP1) whose activity is decreased in cancers. Consequently, increasing LPP1 levels suppresses breast tumor growth and metastasis. This study shows that increasing LPP1 in breast cancer cells decreases transcription through cFos and cJun. This decreases production of cyclin D1/D3 and matrix metalloproteinases (MMPs), which provides new insights into the role of LPP1 in controlling tumor growth and metastasis.

**Methods**: Invasiveness was determined by a Matrigel invasion assay. MMP expression was measured by qPCR, multiplex LASER bead technology and gelatin zymography. Levels of cJUN, cFOS, FRA1, cyclin D1, and cyclin D3 were determined by qPCR and western blotting. Collagen was determined by Picro-Sirius Red staining.

**Results**: Increasing LPP1 expression inhibited invasion of MDA-MB-231 breast cancer cells through Matrigel. This was accompanied by decreases in expression of MMP-1, -3, -7, -9, -10, -12 and -13, which are transcriptionally regulated by the AP-1 complex. Increasing LPP1 attenuated the induction of mRNA of MMP-1, -3, cFOS, and cJUN by EGF or TNFα, but increased FRA1. LPP1 expression also decreased the induction of protein levels for cFOS and cJUN in nuclei and cytoplasmic fractions by EGF and TNFα. Protein levels of cyclin D1 and D3 were also decreased by LPP1. Although FRA1 in total cell lysates or cytoplasm was increased by LPP1, nuclear FRA1 was not affected. LPP1-induced decreases in MMPs in mouse tumors created with MDA-MB-231 cells were accompanied by increased collagen in the tumors and fewer lung metastases. Knockdown of LPP1 in MDA-MB-231 cells increased the protein levels of MMP-1 and -3. Human breast tumors also have lower levels of LPP1 and higher levels of cJUN, cFOS, MMP-1, -7, -8, -9, -12, -13, cyclin D1, and cyclin D3 relative to normal breast tissue.

**Conclusion**: This study demonstrated that the low LPP1 expression in breast cancer cells is associated with high levels of cyclin D1/D3 and MMPs as a result of increased transcription by cFOS and cJUN. Increasing LPP1 expression provides a novel approach for decreasing transcription through AP-1, which could provide a strategy for decreasing tumor growth and metastasis.

## Introduction

Tumor metastasis is the leading cause of mortality in breast cancer patients and therapeutic approaches to control metastasis are largely ineffective. Lysophosphatidate (LPA) is a lipid mediator that stimulates tumor growth and metastasis by signaling through G protein-coupled receptors (GPCRs) 1-3. High levels of LPA and LPA1, -2 and -3 receptors are reported in multiple types of cancers including breast and ovarian cancer 4, 5. Consequently, blocking LPA signaling in breast cancer showed therapeutic benefits in decreasing tumor growth and metastasis in preclinical studies 6, 7.

Another strategy to inhibit excessive LPA signaling in cancers is by increasing the levels of lipid phosphate phosphatases (LPPs). These are a family of three enzymes (LPP1, -2, and -3) that hydrolyze LPA and other bioactive lipid phosphates and pyrophosphates 2, 8. Significantly, the expressions of LPP1 (*PLPP1*) and LPP3 (*PLPP3*) are decreased in lung, ovarian and breast tumors, whereas that of LPP2 (*PLPP2*) is increased 8. We increased the low LPP1 expression in breast cancer cells and showed that this suppresses tumor growth and metastasis by ~80% in syngeneic and xenograft mouse models 6. Overexpression of LPP3 or LPP1 in ovarian cancer cells had a similar effect 9, 10.

LPP1, -2, and -3 are located partly on the plasma membrane where the catalytic sites are on the outer surface 11. Thus, the LPPs are “ecto-phosphatases” that degrade extracellular LPA 8. Consequently, part of the tumor-suppressing activity of LPP1 is accounted for by increased LPA degradation and decreased LPA signaling through its receptors 6. In addition, there is increasing evidence for the intracellular functions of LPP1 that occur downstream of the activation of GPCRs. For example, increasing LPP1 expression decreased the effects of wls-31 in activating of cell migration through phospholipase D in fibroblasts 12 and through activation of Ca^2+^ transients in MDA-MB-231 breast cancer cells 6. Wls-31 is a phosphonate analog of LPA that activates LPA_1/2_ receptors but which cannot be dephosphorylated by LPP1. LPP1 also inhibited the activation of ERK 13 and Ca^2+^ transients 6 by thrombin or protease-activated receptor-1 (PAR-1), respectively. These latter effects of LPP1 indicate that it attenuates signaling downstream of the activation of GPCRs and that these actions contribute to the complex mechanisms through which LPP1 suppresses tumor growth and metastasis.

The capacity of cancer cells to escape from the primary tumor, move into lymphatic and blood vessels and then invade other tissues is facilitated by matrix metalloproteinases (MMPs) of which one of the functions is degrading the extracellular matrix (ECM) 14. MMPs constitute a family of zinc-dependent endopeptidases with more than 20 members, including collagenases (MMP-1, -8, -13, -18), gelatinases (MMP-2, -9), stromelysins (MMP-3, -10, -11), matrilysins (MMP-7, -26), membrane-type MMPs, and other non-classified MMPs 15, 16. Increased expression of MMPs is well documented in breast tumors and this is significantly associated with tumor aggressiveness and poor prognosis 17-19. Expression of several MMPs is regulated by a variety of transcription factors such as activator protein 1 (AP-1), E26 transformation-specific transcription factors (ETS), specificity protein 1 (Sp1) and nuclear factor-κB (NFĸB) 20, 21 through binding to their response elements in the promoter regions. AP-1 is a family of basic leucine-zipper (bZIP) transcription factors that generally bind DNA as heterodimers composed of one member each of the FOS family (cFOS, FRA1, FRA2 and FOSB) and the JUN family (cJUN, JUNB and JUND) 22. AP-1 binding sites exist in the promoters of MMP-1, -2, -3, -7, -9, -10, -12, -13, -19, and -26 21, 23-25. Regulation of MMPs by AP-1 has been demonstrated in MDA-MB-231 breast cancer cells and U-2 OS osteosarcoma cells 26-28. Decreasing cJUN and FRA1 levels in MDA-MB-231 cells inhibited MMP-2 and -9 expression and suppressed tumor metastasis in mice 26. AP-1 also regulates the production of cyclin D1 and D3, which are required for progression through the G1 to S phase of the cells cycle. In addition, cyclin D1 29, 30 and cyclin D3 31 play an important role in cell invasiveness and their expressions are associated with increased metastasis in several types of cancer.

Little is known about whether LPP1 affects the expressions of MMPs in breast cancer. Several studies reported that LPA increases the expression of MMP-2 or MMP-9 in leukemic monocytes 32, neuroblastoma 33, hepatocellular carcinoma cells 34, and ovarian cancer cells 35. This suggests that the low LPP1 activity in cancers could increase MMP levels through the concomitant elevation in LPA signaling. We also have no information about the role of LPP1 in regulation transcription through AP-1 and how this might change the expression of cyclin D1/3. The present study is the first demonstration that the low LPP1 activity in breast cancer cells leads to increased production of MMPs and cyclin D1/3 by transcriptional regulation through the AP-1 complex. This action can contribute to tumor metastasis and the decreased survival of patients who have very low LPP1 expression in their tumors. Increasing LPP1 expression provides a novel and promising strategy for suppressing tumor progression and metastasis.

## Methods

### Reagents and antibodies

Recombinant human EGF (CRE009B) was from Cell Sciences (Canton, MA). Recombinant human TNFα (Z100855), protease inhibitor cocktail (G135), reverse transcription master mix (G490) and EvaGreen qPCR master mix (MasterMix-ER) were from Applied Biological Materials Inc. (Vancouver, BC, Canada). Growth factor-reduced Matrigel (3433-005-01) was from R&D Systems (Minneapolis, MN). Six-well transwells with 8.0 µm pore polycarbonate membrane inserts (3428) were from Corning (Pittsburgh, PA). Sirius red (365548) and MEK inhibitor PD98059 (513000) were from Millipore Sigma (Burlington, MA). P38 MAPK inhibitor SB202190 (10010399) was from Cayman Chemical Company (Ann Arbor, MI). JNK inhibitor SP600125 (1496) was from Bio-Techne Corporation (Minneapolis, MN). Mouse anti-c-Myc (sc-40) antibody was from Santa Cruz Biotechnology (Dallas, TX). Rabbit anti-cyclin D1 (#2922), mouse anti-cyclin D3 (#2936), rabbit anti-cJUN (#9165), rabbit anti-FRA1 (#5281) and rabbit anti-cFOS (#4384) antibodies were from Cell Signaling Technology (Danvers, MA). pENTR/D-TOPO cloning kit (K240020), LR clonase enzyme mix (11791019), siRNA for human LPP1 (AM51331, AM16708) and negative control siRNA (AM4611) were from ThermoFisher Scientific (Grand Island, NY). TransIT-BrCa (MIR5504) transfection reagent was from Mirus Bio, (Madison, WI). Prime-Fect (20-10) for siRNA transfection was from RJH Biosciences (Edmonton, AB, Canada). PfuUltra DNA polymerase (600385) was from Agilent Technologies (Santa Clara, CA).

### Patient and cell line data analysis

Tumor samples were taken from 72 breast cancer patients during surgery at the University of Alberta Hospital. Normal breast tissues as control were taken from 12 patients receiving breast reduction surgery. The procedure was approved by the Ethics Committee of University of Alberta. Microarray mRNA data were obtained from The Cancer Genome Atlas (TCGA) containing 817 breast cancer patients 36 or Cancer Cell Line Encyclopedia (CCLE) of the Broad Institute and Novartis containing 56 types of breast cancer cell lines 37 through the website of cBioPortal (www.cbioportal.org). LPP1 mRNA level is categorized into “high” (z-score > 0) or “low” (z-score < 0) relative to the average of the reference population based on the z-score. The disease-free survival was analyzed with Gehan-Breslow-Wilcoxon test, and the correlations of gene expression were analyzed by linear regression.

### Construct preparation, lentivirus infection and treatment of cells

Human LPP1 with a myc-tag at the C-terminus was generated by PCR using cDNA of HEK293 cells. Mouse LPP1 was subcloned from the pEN_TTmcs-mLPP1 vector that we made previously 6. They were firstly cloned into the pENTR/D-TOPO vector by TOPO reaction, and then transferred into the pLenti-PGK-Neo-DEST vector (19067 Addgene) carrying a neomycin selection marker through LR recombination. Lentivirus was generated as described previously 8 by co-transfecting the lentiviral vector and packaging vectors into HEK293T cells. MDA-MB-231 and 4T1 cells were from ATCC (Manassas, VA) and cultured in DMEM supplemented with 10% FBS. BT-549 cells were a gift from Dr. Roger Leng (University of Alberta, Canada), and cultured in RPMI with 10% FBS and 0.023 IU/ml insulin. Cells stably expressing myc-tagged LPP1 were established by selection with G418 (Geneticin) after lentivirus infection. Cells infected by empty lentivirus were used as control.

Sequences of cFOS, FRA1, and cJUN were transferred into pEZY3 (18672, Addgene) from pDONR221-FOS (HsCD00045461, DNASU), pDONR221-FOSL1 (HsCD00045349, DNASU) and pDONR-JUN (82138, Addgene) through LR reaction to generate plasmids coding cFOS, FRA1, and cJUN respectively.

To determine the effect of MAPK cascades on MMP mRNA expression, cells were treated for 24 h with 10 μM SP600125, 20 μM PD98059 or 5 μM SB202190 to inhibit JNK, MEK and p38 kinase, respectively. Control cells were treated with the same volume of DMSO. To determine the effect of AP-1 on MMP expression, cells in 12-well plates were transfect with 1 μg plasmid for cJUN, cFOS, or FRA1. Protein or mRNA was extracted at 48 h after transfection.

To determine EGF- or TNFα-induced phosphorylation of JNK, ERK and p38, cells were cultured to 80% confluence in 12-well plates and serum-starved overnight in DMEM with 0.1% BSA. Cells were stimulated with 100 ng/ml EGF or 50 ng/ml TNFα for different times, and the stimulation was stopped by washing cells with cold PBS. Cells were lysed with RIPA buffer containing protease inhibitor cocktail and phosphatase inhibitors. Protein concentrations of the cell lysates were determined by the BCA method and lysates were adjusted to the same protein level for Western blotting as described previously 6.

### Matrigel invasion assay

Matrigel invasion assays were performed using 6-well Transwell inserts (Costar, #3428) coated with 300 μl of 1.2 mg/ml Matrigel diluted with starvation media (serum-free DMEM containing 0.1% BSA). Two ml of cells (5 × 10^4^/ml) were suspended in the starvation media and seeded on the top of the layer of Matrigel in the upper chambers. The lower chambers were filled with 2 ml DMEM with 10% FBS. The inserts were incubated in a tissue culture incubator (37°C, 5% CO_2_ and 95% relative humidity) for 12 h. After incubation, cells remaining in the upper chamber were wiped away with cotton swabs and cells that invaded and migrated through the Matrigel-coated membrane were fixed with cold methanol and stained with crystal violet solution (0.5 g crystal violet, 80 ml H_2_O and 20 ml methanol). Inserts were washed three times with PBS and the cell-attaching membranes were cut out. Crystal violet was extracted with 1 ml 10% acetic acid and OD_590nm_ was measured.

### Measurement of MMP concentrations

MMP concentrations in the conditioned media of cancer cells and in tumors were measured by Eve Technologies Corp. (Calgary, AB, Canada) using multiplexing LASER bead technology on a Luminex 100 system (Luminex, Austin, TX). For measuring MMPs in conditioned medium, cells at 80-90% confluence were cultured in 6-well plates using 1 ml DMEM with 0.1% BSA for 24 h. The volumes of the conditioned medium were adjusted according to the protein content of cells from the same wells. For measuring MMP concentrations in tumor tissue, tumors were homogenized with RIPA buffer containing protease inhibitor cocktail. Supernatants were collected after centrifugation at 12000 g for 30 min at 4°C. The protein levels were determined by the BCA method and the supernatants were adjusted to the same protein concentration.

### Gelatin zymography

Cells in 6-well plates were cultured to 80-90% confluence before the experiment. For preparing conditioned medium, cells were cultured in 1 ml of FBS- and BSA-free DMEM for 24h. Conditioned medium was centrifuged (12000 g, 4 °C) through a 10 kDa cut-off protein filter and concentrated by ~10 times. The final volume of the conditioned medium was adjusted according to the protein content of cells from the same wells. Conditioned medium was mixed with 4× sample buffer (0.25M Tris/HCl, pH 6.8, 40% v/v glycerol, 8% SDS and 0.01% bromophenol blue) without heating, and resolved by a 10% polyacrylamide gel containing 0.1% gelatin. Gels were washed with 2.5% Triton-X100 and rinsed with water. Gels were incubated in developing buffer (50 mM Tris/HCl, pH 7.8, 5 mM CaCl_2_, 200 mM NaCl, 0.2% Brij 35) at 37°C for 16 h, and stained with 0.05% Coomassie Blue. After destaining, the gels were scanned and analyzed with Odyssey infrared imaging system (LI-COR Biosciences, Lincoln, NE).

### Nucleocytoplasmic partitioning

MDA-MB-231 cells were cultured to 80% confluence in 10-cm dishes and serum-starved over night. Cells were stimulated with 100 ng/ml of EGF or 50 ng/ml TNFα for 1 and 2 h. Cells were washed twice with ice-cold PBS followed by adding 0.25 ml of lysis buffer (10 mM HEPES, pH 7.5, 10 mM KCl, 0.1 mM EDTA, 0.5% Nonidet-P40 and protease inhibitor cocktail). Cells were scrapped and kept on ice for 30 min. After centrifugation at 12,000 g for 10 min at 4°C, supernatants were collected as the cytoplasm fraction. Pellets were washed 3 times with lysis buffer and resuspended and sonicated in 0.25 ml RIPA buffer. After centrifugation at 12,000 g for 10 min at 4°C, the supernatant was collected as the nuclear fraction. Proteins of interest were determined by Western blotting and analyzed with Odyssey infrared imaging system (LI-COR Biosciences, Lincoln, NE).

### Real-time PCR and western blotting

MMP-1, MMP-3, MMP-13, cJUN, cFOS, and FRA1 mRNA levels were determined by real-time PCR after reverse transcription 6. GAPDH was used as a reference gene. Primer sequences were listed in Table 1. Protein levels were measured by western blotting as described previously 6. Immunoblots were analyzed by Odyssey infrared imaging system (LI-COR Biosciences, Lincoln, NE).

### Mouse model of breast cancer

Female NSG (NOD scid gamma) mice at 12 weeks of age were purchased from the breeding colony of Dr. Lynne Postovit (University of Alberta, Canada). Xenograft orthotopic mouse breast cancer models were established by inoculating MDA-MB-231 cells into the mammary fat pads of the mice as described previously 6. Tumor growth was monitored by measuring the length (L) and width (W) of the tumor with caliper. Tumor volume was calculated using the formula L×W^2^/2. After sacrificing the mice (n=8 mice per group), the tumors were excised carefully and weighed. Lungs were fixed by injecting 10% formalin into the trachea. Part of the lungs (n=5 per group) were stained with India ink. Visible lung nodules were counted. All procedures were performed in accordance with the Canadian Council of Animal Care as approved by the University of Alberta Animal Welfare Committee.

### Picro-Sirius Red staining and H&E staining

Tumors and part of the lungs were fixed with formalin followed with paraffin embedding and sectioning. Tumor sections were staining by 0.1% Sirius red solution (0.5g Sirius red dissolved in saturated picric acid water solution) for 1 h and washed twice by 0.5% acetic acid. For each sample, enough images were taken by a Zeiss Axioskop 2 imaging system (Carl Zeiss Canada, Toronto, ON, Canada) to cover the entire tissue area. Collagen fibers stained as bright red were picked out and the relative areas of collagen fibers were analyzed by ImageJ software 38. The average value for each sample was calculated from all the images for that sample. H&E staining was performed according to the method reported previously 6.

### Statistical analysis

Results were analyzed with a student t-test or by ANOVA for multiple comparisons followed by Newman-Keuls test. *P*<0.05 was considered as statistically significant.

## Results

### Increasing LPP1 expression in breast cancer cells decreased MMP expression and inhibited invasion

**Figure 1:**
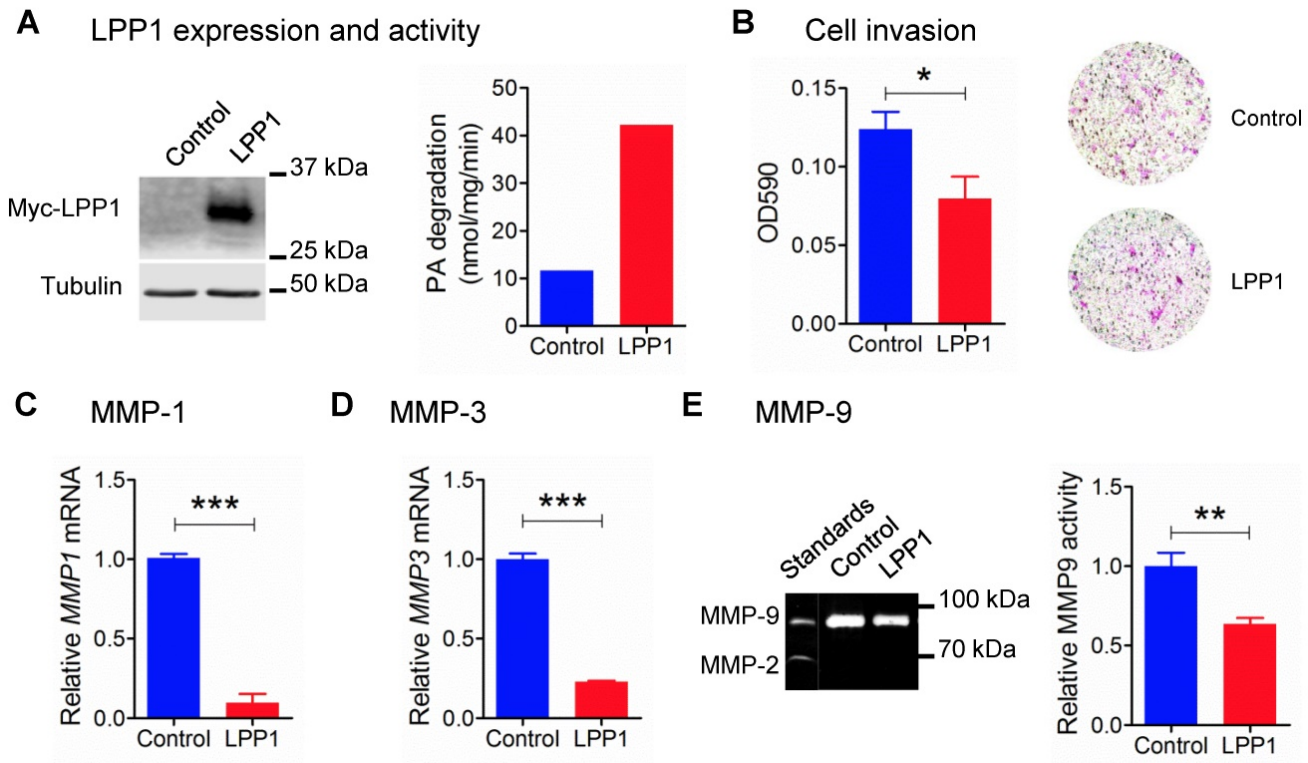
** A:** Protein expression of myc-tagged LPP1 and total LPP activity in MDA-MB-231 cells stably expressing LPP1. **B:** Increased LPP1 expression inhibited MDA-MB-231 cell invasion through Matrigel. Cells invade through the Matrigel were fixed and stained with crystal violet as shown by the representative images. **C** and **D:** Increased LPP1 expression significantly suppressed mRNA levels of MMP-1 and MMP-3 in MDA-MB-231 cells. **E:** Increased LPP1 expression decreased MMP-9 activity in MDA-MB-231 cells as measured by zymography. Results are means and SEM from three independent experiments. Results were analyzed by Student's *t*-test, *P < 0.05, ** *P*<0.01, *** *P* < 0.001.

Our previous work showed that increasing the low LPP1 expression in breast cancer cells decreases lung metastasis in syngeneic and xenograft mouse breast cancer models using 4T1 and MDA-MB-231 cells, respectively 6. Therefore, we examined if LPP1 affects cancer cell invasion, which is a major component of metastasis. Stable expression of myc-tagged LPP1 in MDA-MB-231 cells increased the total LPP activity by ~3.6 fold (Figure 1A), and this inhibited cell invasion through Matrigel by ~36% (Figure 1B). We, therefore, hypothesized that LPP1 could modify interactions with the extracellular matrix through modification of the expression of MMPs. We measured this by studying selected MMPs and showed that increasing LPP1 activity decreased mRNA for MMP-1 and MMP-3 by 91% and 77%, respectively (Figure 1C and D). MMP-9 activity as detected by zymography was deceased by ~36% (Figure 1E). Overexpressing LPP1 also decreased mRNA levels of MMP-1, MMP-3 and MMP-13 in BT-549 breast cancer cells (Supplementary Figure 1A) and 4T1 mouse breast cancer cells (Supplementary Figure 1B).

### LPP1 decreased the expression of MMP-1 and -3 through regulation of AP-1

**Figure 2:**
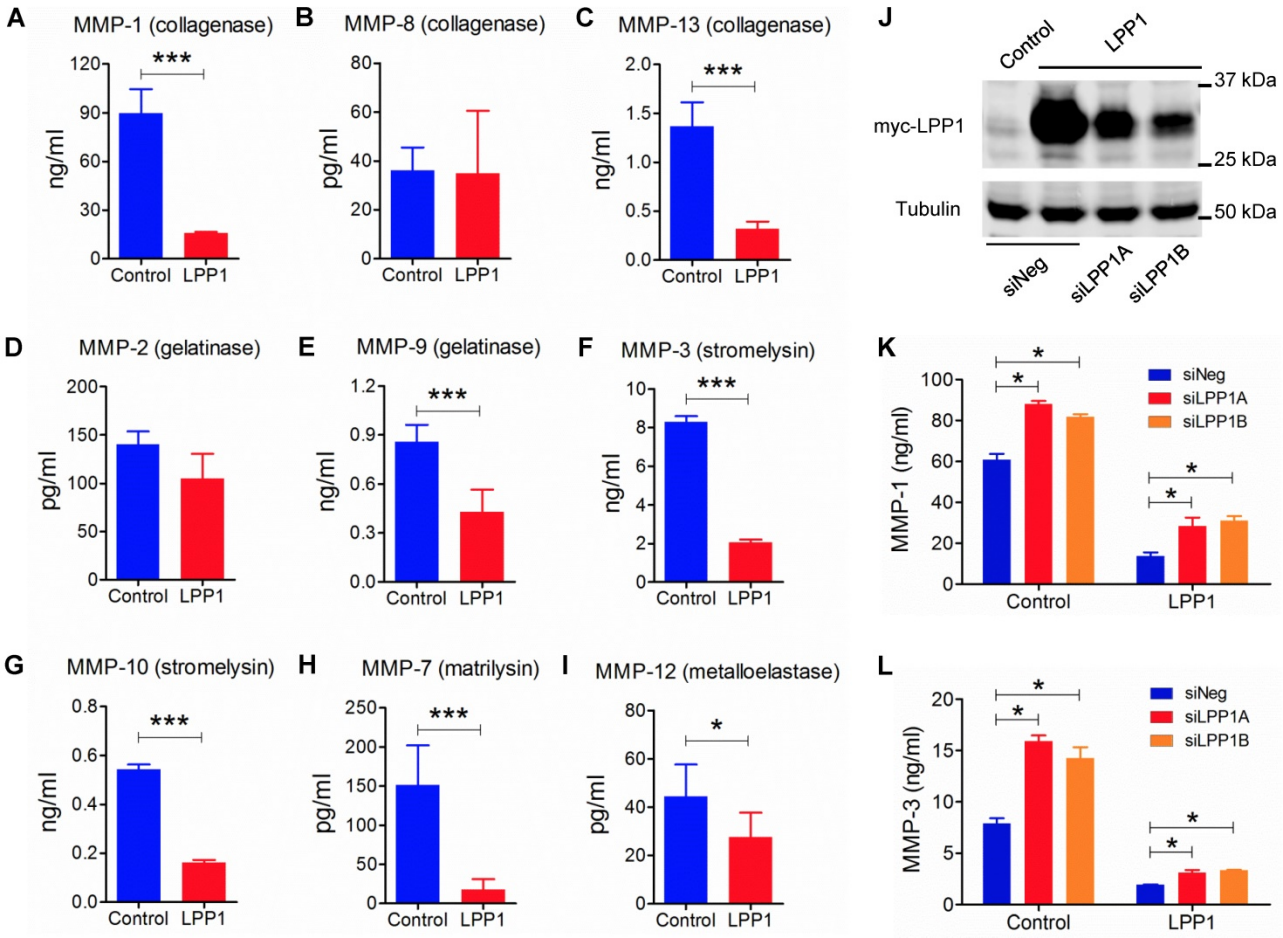
** A - I:** Increased LPP1 expression decreased the concentrations of collagenases (MMP-1, -13), gelatinase (MMP-9), stromelysins (MMP-3, -10), matrilysin (MMP-7), and metalloelastase (MMP-12) in the conditioned media from MDA-MB-231 cells. **J:** Expression of myc-tagged LPP1 in MDA-MB-231 cells was knocked down by two sets of siRNA for LPP1 (siLPP1A, siLPP1B). **K** and **L:** Knockdown of LPP1 increased the concentrations of MMP-1 and MMP-3 in the conditioned media from MDA-MB-231 cells, and partially reversed the decrease in MMP-1 and MMP-3 mediated by LPP1. Results are means and SEM, and analyzed by Student's *t*-test or ANOVA, n=6, *P < 0.05, *** *P* < 0.001.

We, therefore, investigated the effect of increasing LPP1 expression on the production of MMP protein in MDA-MB-231 cells. Within the nine MMPs that were measured, MMP-1 (89.7 ± 6.0 ng/ml) had the highest concentration and this was decreased by 82% in the conditioned media by increasing LPP1 activity (Figure 2). Concentrations of MMP-13 (collagenase), -9 (gelatinase), -3 (stromelysin), -10 (stromelysin), -7 (matrilysin) and -12 (metalloelastase) were lower than 9.0 ng/ml. Increasing LPP1 activity decreased these concentrations by 82%, 77%, 50%, 75%, 70%, 88% and 38%, respectively (Figures 2 A - I). Concentrations of MMP-1 and -3 were also decreased in BT-549 cells by increasing LPP1 expression (Supplementary Figure 1A). Conversely, LPP1 knockdown with two different siRNA increased MMP-1 and -3 concentrations and partially reversed the LPP1-mediated suppression of MMP-1 and -3 in the conditioned media of MDA-MB-231 cells (Figures 2 J - L). LPP1 knockdown also increased concentrations of MMP-7, -9, -10 and -13 but did not reverse the inhibitory effects of LPP1 on these MMPs (Supplementary Figure 2).

**Figure 3:**
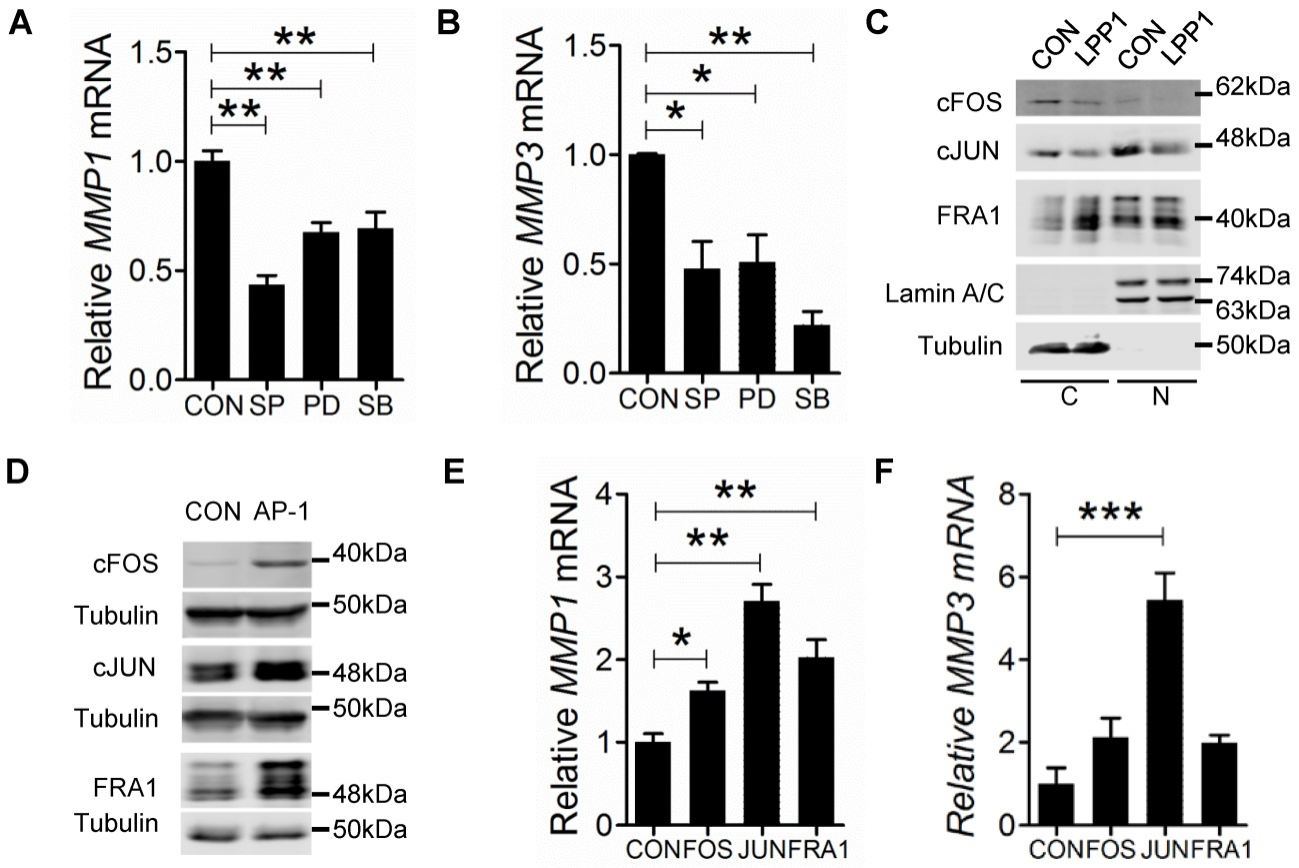
** A** and **B:** MDA-MB-231 cells treated with 10 μM JNK inhibitor SP 600125 (SP), 20 μM MEK inhibitor PD 98059 (PD) or 5 μM p38 inhibitor SB 202190 (SB) for 24 h showed significantly decreased mRNA levels of MMP-1 and MMP-3 relative to the control cells (CON). **C:** MDA-MB-231 cells with increased LPP1 expression showed decreased cFOS and cJUN in cytoplasm (C) and nuclei (N) compared with control cells (CON). The level of FRA1 was increased in cytoplasm but not affected in nuclei by LPP1. **D:** Protein levels of cFOS, cJUN, or FRA1 in MDA-MB-231 cells overexpressing these proteins (AP-1) and in control cells (CON). **E a**nd **F:** Transient expressions of cFOS, cJUN or FRA1 significantly increased mRNA levels of MMP-1 and MMP-3 in MDA-MB-231 cells. Results are means and SEM from three independent experiments. Results were analyzed by ANOVA. *P < 0.05, ** *P* < 0.01, *** *P*<0.001.

The MMPs that were decreased by LPP1 expression all have AP-1 binding sites in the promoters of their genes 21. Activation of AP-1 in response to a diverse array of extracellular stimuli relies on phosphorylation of MAPKs 39. We, therefore, tested the effects of inhibitors for MEK (PD98059, 20 μM), p38 (SB202190, 5 μM) or JNK (SP600125, 10 μM), which are kinases of the MAPK cascades. These inhibitors significantly decreased mRNA expression of MMP-1 and -3 in MDA-MB-231 cells (Figures 3A and B). LPP1 did not affect phosphorylation of JNK, ERK and p38 in response to stimulation with TNFα or EGF (Supplementary Figure 3A and B), suggesting that LPP1 may affect signaling downstream of the MAPK cascades.

We then determined whether LPP1 affects protein levels of cJUN, cFOS, or FRA1. Increasing LPP1 expression decreased cJUN and cFOS in both cytoplasm and nuclei (Figure 3C). FRA1 was increased by LPP1 in the cytoplasm, but the nuclear FRA1 was not affected (Figure 3C). We checked the significance of this result using the CCLE dataset containing 56 breast cancer cell lines and showed that expression of LPP1 was negatively correlated with cFOS (Supplementary Figure 4A) and positively correlated with FRA1 (Supplementary Figure 4C), which is in agreement with our results. There was no correlation between LPP1 and cJUN in the dataset (Supplementary Figure 4B). To further establish this relationship, we showed that MMP-1 mRNA was significantly increased by transient expression of cFOS, cJUN or FRA1 in MDA-MB-231 cells, and MMP-3 mRNA was significantly increased by cJUN (Figures 3D, E, and F).

### LPP1 inhibited EGF-induced expression of cFOS and cJUN but not FRA1

**Figure 4:**
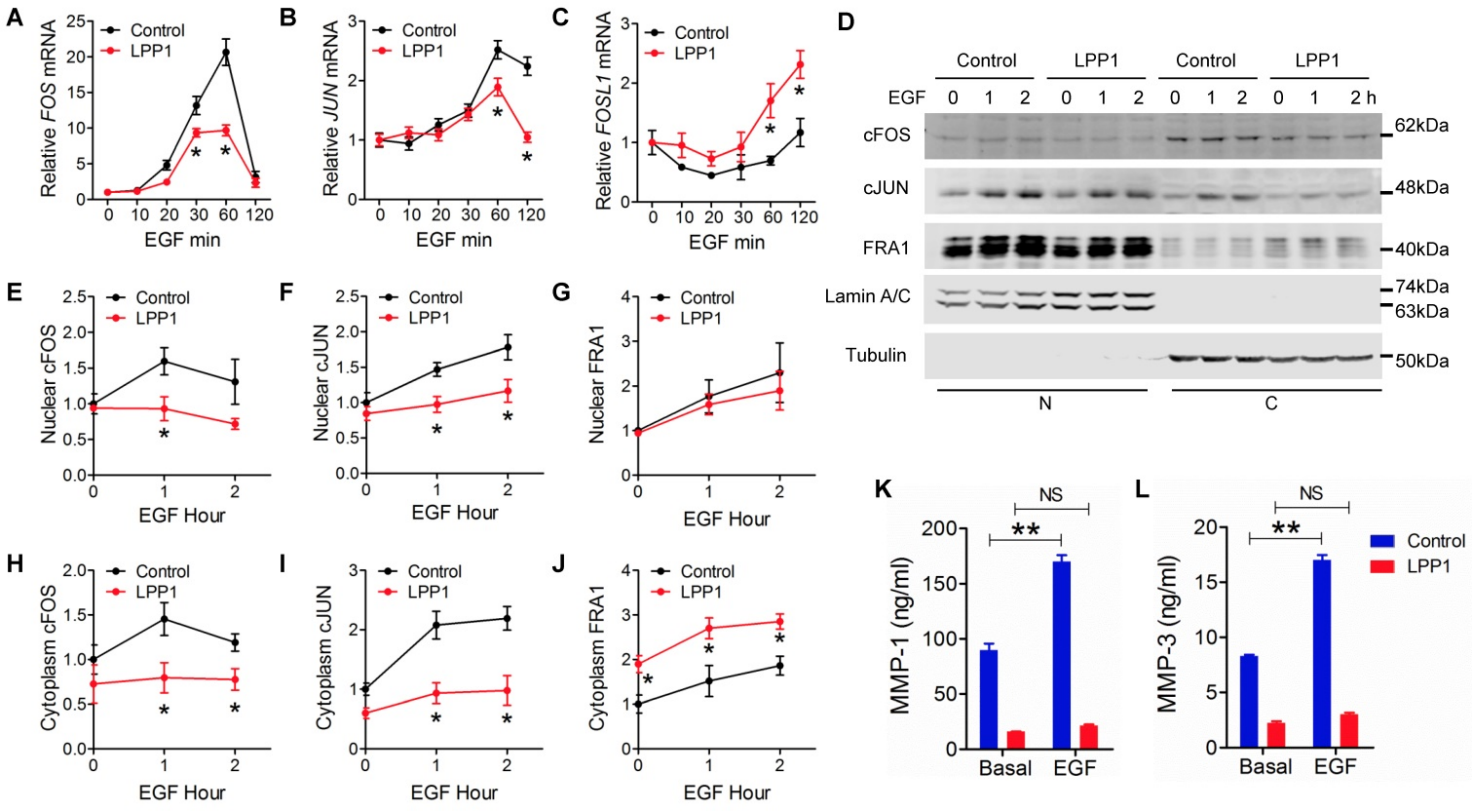
** A - C:** EGF (100 ng/ml) induced mRNA expression of cFOS (*FOS*), cJUN (*JUN*) and FRA1 (*FOSL1*) in MDA-MB-231 cells with or without increased expression of LPP1. **D:** EGF (100 ng/ml) induced expression of cFOS, cJUN and FRA1 in nuclei (N) and cytoplasm (C) from MDA-MB-231 cells with or without increased expression of LPP1. **E - J:** Quantification for EGF-induced cFOS (E and H), cJUN (F and I) and FRA1 (G and J) from panel **D**. **K** and **L:** EGF-induced increases in concentrations of MMP-1 and MMP-3 in the conditioned media of MDA-MB-231 cells were suppressed by LPP1. Results are means and SEM from three independent experiments. Results were analyzed by ANOVA. *P < 0.05 relative to control, ** *P* < 0.01, NS: not significant.

We then determined the effects of LPP1 on EGF-induced expression of cFOS (*FOS*), cJUN (*JUN*), and FRA1 (*FOSL1*). EGF increased cFOS and cJUN mRNA with a peak at 60 min after stimulation. This was suppressed by LPP1 (Figure 4A and B). Stimulation by EGF after 2 h did not significantly increase FRA1 mRNA in control cells, but significantly increased it in LPP1 expressing cells (Figure 4C).

As transcription factors, cFOS, cJUN and FRA1 exert their functions in nuclei. EGF stimulation caused increases in nuclear cFOS, cJUN and FRA1 in MDA-MB-231 cells. LPP1 decreased the induction of cFOS and cJUN, but not FRA1 in nuclei (Figures 4 D - G). Cells with increased LPP1 activity also showed less cFOS and cJUN and more FRA1 in cytoplasm after EGF stimulation relative to control (Figure 4D, H, I, and J). Accordingly, EGF-induced secretions of MMP-1 and -3 in the conditioned media were decreased by LPP1 expression (Figures 4K and L). These results provide evidence that the decreases in nuclear cFOS and cJUN could account for the decrease in MMP production. Although total FRA1 was increased by LPP1, it accumulated mainly in the cytoplasm and the amount in nuclei was not changed. Therefore, the change of FRA1 does not correlate with the inhibition in MMP production by LPP1. Stimulation of the MDA-MB-231 cells with TNFα showed similar effects on the expression of cFOS, cJUN and FRA1 as EGF (Supplementary Figure 5).

**Figure 5:**
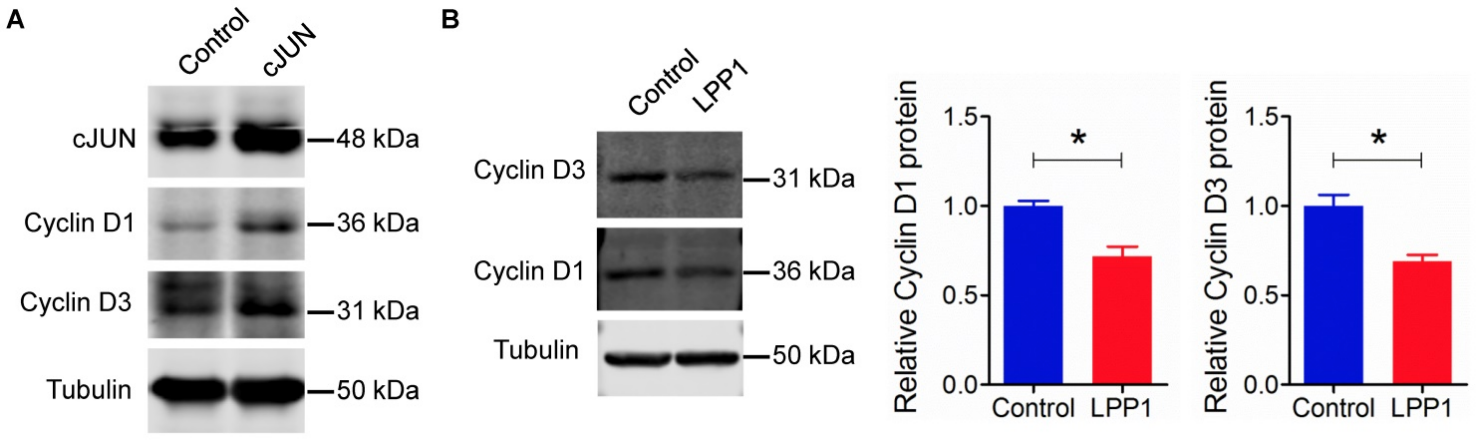
** A:** Increased expression of cJUN increased the levels of cyclin D1 and D3 in MDA-MB-231 cells. **B:** Increased expression of LPP1 decreased the levels of cyclin D1 and D3 in MDA-MB-231 cells. Results are means and SEM from three independent experiments. Results were analyzed by Student's *t*-test. *P<0.05.

AP-1 also regulates the expression of other genes, for example, cyclin D1 40. Overexpression of cJUN in MDA-MB-231 cells increased the levels of cyclin D1 and cyclin D3 (Figure 5A). Increasing LPP1 activity decreased cyclin D1 and D3 by ~28% and 31% respectively (Figure 5B), which further confirmed the inhibition of AP-1 by LPP1 expression.

Treatment by 5 μM LPA or sphingosine-1-phosphate (S1P) for 24 h did not affect the secretion of MMPs (results not shown) in MDA-MB-231 cells, suggesting that LPA or S1P signaling does not regulate MMP expression under these conditions. LPP1 did not affect EGF-induced phosphorylation of ERK (Supplementary Figure 2B) and Akt 6, indicating that the function of EGF receptor was not impaired by LPP1. MMPs can also be induced by NFκB 20, 21 and inflammatory cytokines 41-43. We showed that LPP1 did not affect TNFα-induced increase of NFκB in nuclei. Phosphorylation of NFκB and degradation of IκB were not significantly changed by LPP1 (Supplementary Figure 6). Cytokine levels in the conditioned media of unstimulated MDA-MB-231 cells were also not changed by LPP1 (Supplementary Figure 7). This result excluded the effect of LPP1 on NFκB function and cytokine-mediated autocrine regulation of MMP expressions.

### MMPs were decreased in breast tumors derived from MDA-MB-231 cells expressing LPP1

**Figure 6:**
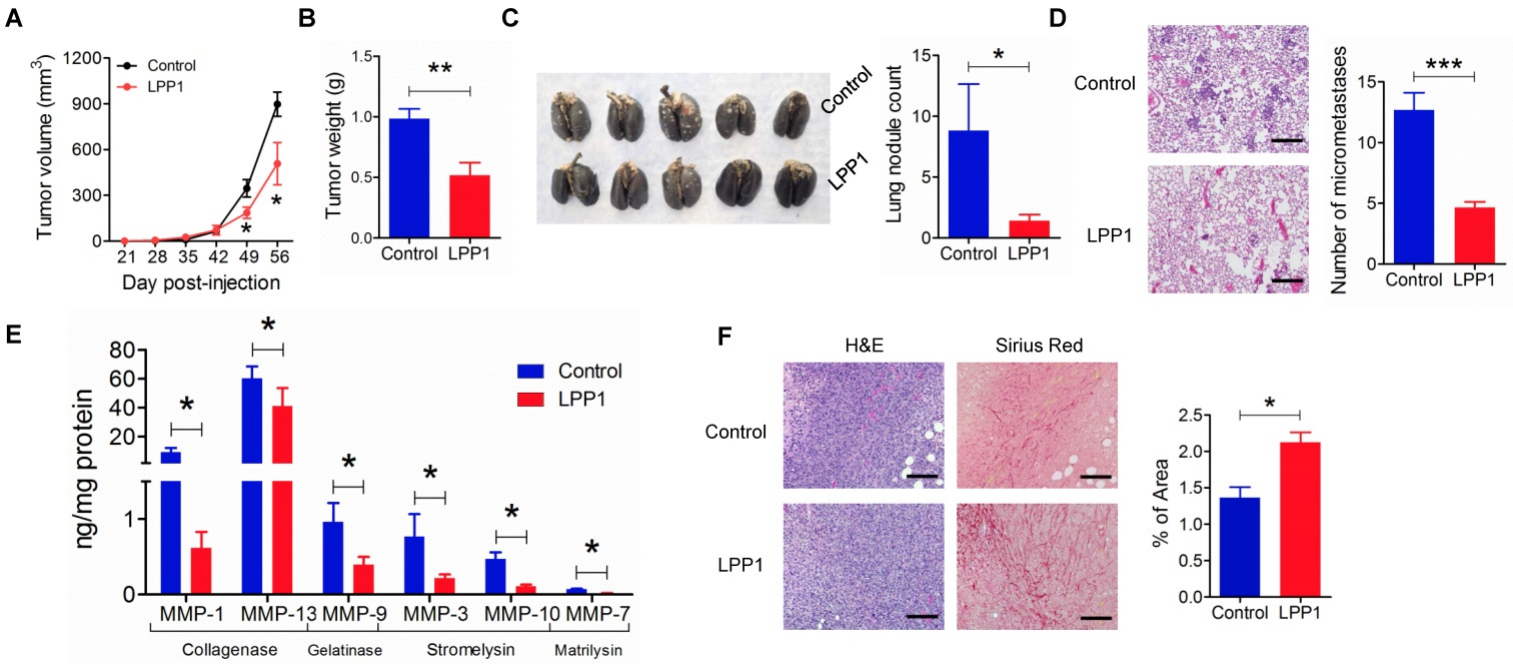
Increasing LPP1 expression in MDA-MB-231 cells decreases breast tumor growth and metastasis with increased collagen content in the tumors. **A:** Tumor size (n=8 per group). **B:** Tumor weight (n=8 per group). **C:** Number of nodules on lung surface (n=5 per group). **D:** Micrometastasis in lungs (n=3 per group). **E:** LPP1 significantly decreased MMPs in the tumors (n=8 per group). **F:** Collagen fibers in tumors detected by Picro-Sirius Red staining (n=8 per group). Images were taken by 10× lens. The scale bar represents 120 μm. Results are means and SEM and analyzed by Student's *t*-test or ANOVA. *P < 0.05, ** *P* < 0.01, *** *P* < 0.001 relative to control.

We next confirmed the role of LPP1 in regulating MMP expressions in a mouse model of breast cancer. The expression of LPP1 in breast cancer cells in our previous work 6 was driven by a doxycycline inducible promoter. This causes interference in studying the MMPs since doxycycline is an MMP inhibitor 44. Therefore, in the present study, we used a constitutive promoter for expression of LPP1 to avoid using doxycycline. Consistent with our previous work 6, MDA-MB-231 cells that stably expressed LPP1 formed smaller tumors (Figures 6A and B), had fewer nodules on the lung surface (Figure 6C) and less lung micro-metastases (Figures 6D). Collagenases MMP-1 (9.0 ± 3.0 ng/mg protein) and MMP-13 (54.6 ± 6.9 ng/mg protein) were two major MMPs detected in the tumors. Increased LPP1 expression significantly decreased the concentrations of MMP-1, -13, -3, -7, -9 and -10 in tumors (Figure 6E). Tumors from cancer cells overexpressing LPP1 showed a significant increase in collagen fibers compared with tumors from control cancer cells (Figure 6F). This is in agreement with the decreases in MMP-1 and -13. These results suggest that collagenases generated from breast cancer cells contribute to the degradation of collagen in the tumor microenvironment.

### Relationship between expression of LPP1, LPP3 and MMPs in human breast tumors

**Figure 7:**
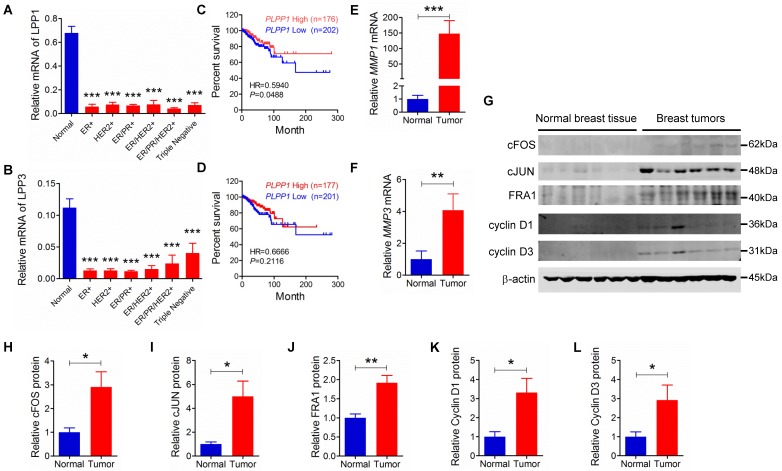
** A** and **B:** Human breast tumors (ER+: n=12, HER2+: n=11, ER/PR+: n=12, ER/HER2+: n=12, ER/HER2/PR+: n=12, triple negative: n=11) had lower mRNA levels for LPP1 and LPP3 relative to normal breast tissue obtained from breast reduction surgery (n=12). Low LPP1 (**C**) but not low LPP3 (**D**) was associated with decreased disease-free survival rate. **E** and **F:** Breast tumors (n=12) had higher MMP-1 and MMP-3 than normal breast tissue (n=12) **G - L:** Breast tumors (n=6) express higher levels of cFOS, cJUN, FRA1, cycline D1, and cyclin D3 than normal breast tissue (n=6). Results are means and SEM and analyzed by Student's *t*-test or ANOVA. The disease-free survival rate was analyzed with Gehan-Breslow-Wilcoxon test. *P < 0.05, ** *P* < 0.01, *** *P* < 0.001.

We extended our work to human breast cancer by determining the relationships between the expressions of LPP1 and MMPs further in our collection of human breast tumors compared to the expressions in 12 samples of normal breast tissue. Unfortunately, existing antibodies for LPP1 and LPP3 are not suitable for detecting the low quantities of these proteins in tumor samples. Our previous work in this area support the use of mRNA for the LPPs as a surrogate marker for their expressions 45, 46. LPP1 and LPP3 mRNA were significantly lower in patient tumors than in normal breast tissue independently of whether the tumors were ER+, HER2+, ER/PR+, ER/PR/HER2+ or triple negative (Figures 7A and B). Furthermore, analysis of the TCGA mRNA microarray dataset, which contains 817 patient samples, showed that breast cancer patients with higher levels of mRNA for LPP1, but not LPP3, have a better disease-free survival rate (Figures 7 C and D). We also determined that MMP-1 and MMP-3 mRNA levels were higher in all types of breast tumors than in normal breast tissue (Figures 7E and F).

**Figure 8:**
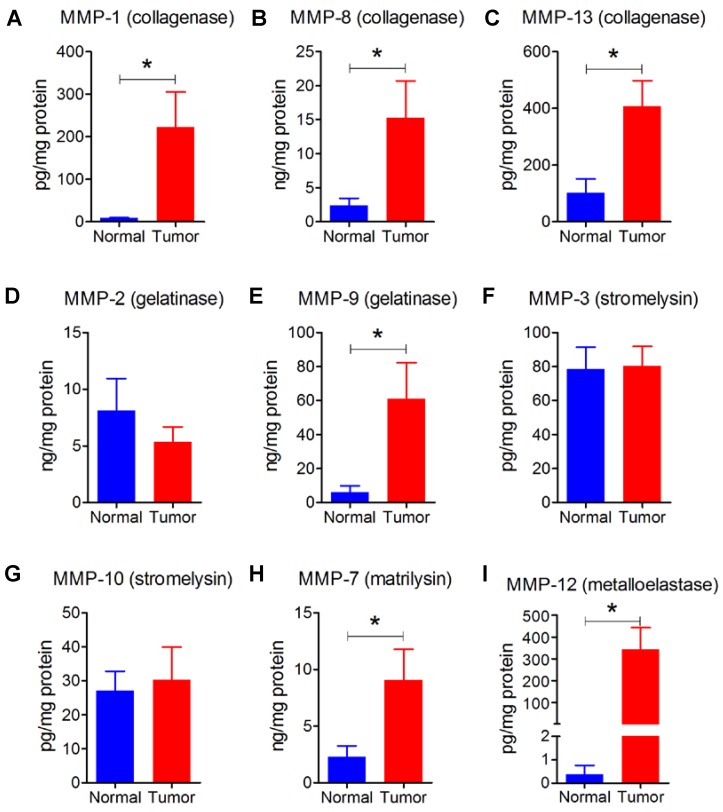
** A - I:** Human tumors (n=6) showed higher levels of collagenases (MMP-1, -8, -13), gelatinase (MMP-9), matrilysin (MMP-7), and metalloelastase (MMP-12) relative to normal breast tissue (n=6). Results are means and SEM and analyzed by Student's *t*-test, *P < 0.05.

We also had six human breast tumors and six normal breast tissue, which had adequate amounts of protein for western blotting and MMP measurement. These human breast tumors had higher content of cFOS, cJUN, FRA1, cyclin D1 and cyclin D3 compared to normal breast tissue (Figures 7G - L). In addition, breast tumors showed higher protein levels of MMP-1, -7, -8, -9, -12, and -13 compared with normal breast tissue (Figure 8). There were no significant differences in the concentrations of MMP-2, MMP-3, and MMP-10.

## Discussion

The present study focused on the role of the LPPs in breast cancer where the expressions of LPP1 and LPP3 are decreased and LPP2 is increased 8. The commercial antibodies for LPPs cannot detect endogenous LPP very well and so we have to use mRNA as a surrogate marker. Low LPP1 expression is associated with a decreased disease-free survival rate compare to those patients with higher LPP1 expression levels. This relationship was not found when comparing lower and higher expression of LPP3. LPP2 has a completely different action to LPP1 and LPP3 in cell signaling since it stimulates cell division 46. LPP2 mRNA was increased as expected 8 by ~7 fold (*P* < 0.001) in our combined breast tumor samples and those patients with higher LPP2 expression had a significantly decreased survival rate compared to those with lower LPP2 levels (results not shown). The mechanisms for the effects of LPP2 are presently unclear, although LPP2 activity is correlated with cell transformation and increases in anchorage-independent cell growth 47. LPPs all dephosphorylate a wide variety of lipid phosphates when assayed *in vitro*, but the substrate specificity is greater *in vivo.* This probably depends on the abilities of the LPPs to access their substrates in different locations in the cell 48. There is substantial evidence that the LPPs have distinct biological functions. For example, knockout of LPP3 in mice results in embryonic lethality 49, whereas mice with KO of LPP2 or hypomorphs for LPP1 are viable 10, 50. Overexpression of Wunen (a homologue of human LPPs) in *Drosophila* causes aberrant migration of primordial germ cells. This situation is mimicked by expression of mammalian LPP3, but not by LPP1 51. These studies reveal specific roles for the LPP isoforms, but the exact mechanisms for these differences are unknown 8, 11, 52.

The present work focused specifically on the role of LPP1 in breast cancer cells where we showed that increasing LPP1 decreases invasion and metastasis. This was related to the effects of LPP1 in decreasing the production of those MMPs that are regulated through AP-1 transcription factors. Our work with breast cancer cells was supported by results obtained from a mouse model of breast cancer and from tumors obtained from breast cancer patients. LPP1 levels were significantly lower in all classes of human breast tumors compared with normal breast tissue. The low LPP1 expression was associated with increased levels of MMPs, cyclin D1/D3, cFOS, cJUN, and FRA1 in breast cancer patients. The increased survival rate in patients with higher LPP1 appears to be a consequence of less metastasis as demonstrated by the lower invasion of breast cancer cells and decreased lung metastasis in a mouse model of breast cancer. Tumor metastasis remains the biggest hurdle for curing breast cancer. Triple negative breast cancer is the most aggressive and metastatic and it lacks effective therapeutic approaches. The low LPP1 level in breast tumors is a promising therapeutic target for treating breast cancer in general including triple negative breast cancer.

We used a tetracycline inducible promoter to increase LPP1 expression in breast cancer cells and this suppressed metastasis 6. This expression system was not suitable for investigating MMPs because tetracycline itself is an MMP inhibitor 45. We, therefore, established breast cancer cells that constitutively express LPP1. The replacement of expression system in this study did not change the effect of LPP1 in suppressing tumor growth and metastasis in mice.

LPA induces the expression of MMP-2 or MMP-9 in a variety types of cancer cells 32-35, which motivated us to investigate the effect of LPP1 on MMPs in breast cancer cells. We did not see changes in MMP concentrations in the conditioned media when we treated MDA-MB-231 cells with 1-10 µM LPA (results not shown). Previous studies also showed that LPA does not affect the expression of MMP-2, -3, and -7 in MDA-MB-231 cells 53. LPA also has no effect on MMP-2 expression in Jurkat cells 54 and CAOV-3 ovarian cancer cells 55. This discrepancy is probably due to the differences in cell lines. We also excluded an indirect autocrine effect of cytokines on MMP expression because LPP1 did not affect the profile of cytokine production in unstimulated MDA-MB-231 cells. The MMPs that were decreased by increasing LPP1 expression in our study all have AP-1 binding sites in their promoter regions 20, suggesting LPP1 may affect the AP-1 signaling pathway. Activation of MAPKs induces the abundance and activity of AP-1 in response to a diverse array of extracellular stimuli, including inflammatory cytokines and growth factors 39. Blocking the MAPK cascades using inhibitors for MEK, p38, and JNK mimicked the effect of LPP1 on MMP-1 and -3 expression. However, LPP1 did not decrease phosphorylation of ERK, p38, and JNK, suggesting that LPP1 may affect the signals downstream of MAPKs, which decreases the levels of cFOS and cJUN. Furthermore, LPP1 suppressed the EGF- or TNFα-induced expression of cFOS and cJUN. These results suggested that the effect of LPP1 on MMPs is independent of the degradation of extracellular LPA. Our previous work indicated that LPP1 not only localized on the plasma membrane 45 but also in the cytoplasm (unpublished results) of MDA-MB-231 cells. Therefore, the intracellular LPP1 may affect signal transduction through AP-1. To our surprise, overexpressing catalytically inactive mutant (R217K) of mouse LPP1 in MDA-MB-231 cells also inhibited the increase in nuclear AP-1 induced by EGF (results not show). This unexpected result suggested the structure of LPP1 instead of its activity may also have an important role in cell signaling.

Some MMPs are also involved in inflammation since they activate PARs 56. PAR activation can in turn stimulate the release of MMPs 57. We already showed that increasing LPP1 expression in breast cancer cells attenuates signaling downstream of PAR receptors 6. AP-1 also regulates cell proliferation, adhesion and cell-cell contact of breast cancer cells 58. AP-1-induced expression of cyclin D1 has been well documented 40, 59. We also found that overexpressing cJUN increases cyclin D1 and D3 in MDA-MB-231 cells. As expected, increasing LPP1 expression decreased the levels of cyclin D1 and D3, and this is most likely due to the decrease in AP-1, which could also contribute the effects of LPP1 expression in decreasing the division and invasiveness of breast cancer cells 6.

MMP production in the tumor microenvironment is regulated by a crosstalk between cancer and stromal cells 60. Cancer cells stimulate the production of stromal-derived MMPs by a paracrine mechanism through secretion of inflammatory cytokines or growth factors 60. MMPs produced by cancer cells can also be induced by stromal cells 61. Our studies only measured the concentration of the MMP proteins, which we needed to relate to AP-1 mediated transcription. The relative concentrations of the MMPs do not necessarily indicate their respective activities since these are regulated by complex covalent modifications of the individual MMPs. The importance of cancer cell-derived MMPs in promoting tumor growth and metastasis has been clearly demonstrated in animal models by overexpression or knockdown of MMPs 62-64. In our mouse model of breast cancer, increasing LPP1 expression in MDA-MB-231 cells decreased multiple MMPs including collagenases, and subsequently, increased in collagen fibers in tumors. These MMPs, which were detected in the mouse tumors were produced by the MDA-MB-231 cancer cells, because the antibodies used in our multiplexing assay are relatively specific for human MMPs. The profile of MMPs in MDA-MB-231 cells grown *in vivo* is different from that *in vitro*, which indicates the tumor microenvironment can modulate MMP production in the cancer cells. For instance, the MMP-13 concentration in tumors was at least ~7 times higher than other MMPs, whereas in the conditioned media form MDA-MD-231 cells, MMP-1 was the most dominant and its concentration was ~66 times higher than MMP-13. This observation is consistent with a previous report 61, which also used MDA-MB-231 cells in a xenograft model of breast cancer. Increasing LPP1 expression in cancer cells attenuated the regulatory effects from stromal cells on MMP-13 production.

These combined results illustrate that increasing the low level of LPP1 expression in breast cancer cells has beneficial effects in decreasing tumor growth and metastasis. We showed that this can be achieved by using tetracycline, which increase the stability of LPP1 45. This results in increased degradation of LPA and decreased LPA signaling, tumor-induced inflammation and decreased breast tumor growth and metastasis 45, 65. Another approach to increasing LPP1 expression is by suppressing inflammation with agents such as dexamethasone, which increases mRNA expression for LPP1 66. Dexamethasone also decreases the production of autotaxin, which produces LPA by breast adipose tissue. These effects of dexamethasone have a comprehensive effect in decreasing the production of a variety of inflammatory cytokines.

## Conclusion

This study demonstrated that decreased LPP1 expression compared to normal breast tissue is a common characteristic of breast tumors irrespective of their classifications. Increasing this low expression of LPP1 in breast cancer cells decreased the invasive capacity of the cancer cells. LPP1 expression also inhibited tumor growth and metastasis, which we related to the decreased production of MMPs and cyclin D1/D3. This was caused by decreasing the levels of cJUN and cFOS in nuclei and it resulted in increased collagen content in the tumors. This study identifies a novel strategy for breast cancer treatment, especially for triple negative breast cancer, which lacks effective therapeutic approaches, by increasing LPP1 expression.

## Supplementary Material

Supplementary figures.

## Figures and Tables

**Table 1:** Primer sequences for real-time PCR.

Genes	Sequence (5'-3')	Accession No.
*MMP1* (human)	F: GCACAAATCCCTTCTACCCG	NG_011740
	R: TGAACAGCCCAGTACTTATTCC	
*MMP3* (human)	F: CCAGGGATTAATGGAGATGCC	NG_012100
	R: AGTGTTGGCTGAGTGAAAGAG	
*MMP3* (mouse)	F: GATGAACGATGGACAGAGGATG	NC_000075
	R: AAACGGGACAAGTCTGTGG	
*MMP13* (mouse)	F: TTGATGCCATTACCAGTCTCC	NC_000075
	R: ACATGGTTGGGAAGTTCTGG	
*JUN* (human)	F: AGCCCAAACTAACCTCACG	NG_047027
	R: TGCTCTGTTTCAGGATCTTGG	
*FOS* (human)	F: TTGTGAAGACCATGACAGGAG	NG_029673
	R: CCATCTTATTCCTTTCCCTTCGG	
*FOSL1* (human)	F: GGGCATGTTCCGAGACTTC	NC_000011
	R: CTCATGGTGTTGATGCTTGG	

## Abbreviations

AP-1: activator protein 1
bZIP: basic leucine-zipper domain
cFOS: cellular FBJ murine osteosarcoma viral oncogene homolog
ECM: extracellular matrix
ERK: extracellular-signal regulated kinase
FRA1: FOS related antigen 1
GPCR: G-protein coupled receptor
JNK: JUN N-terminal kinase
LPA: lysophosphatidate
LPP1: lipid phosphate phosphatase-1
MAPK: mitogen-activated protein kinase
MEK: MAPK/ERK kinase
MMP: matrix metalloproteinase
PAR: protease-activated receptor
S1P: sphingosine 1-phosphate
TNFα: tumor necrosis factor α
