# Supplementary material for: Increasing the low lipid phosphate phosphatase 1 activity in breast cancer cells decreases transcription by AP-1 and expressions of matrix metalloproteinases and cyclin D1/D3
Source: Theranostics. 2019 Aug 14;9(21):6129–42. doi: 10.7150/thno.37094 (PMC6735510; doi:10.7150/thno.37094)
Supplement: Supplementary file 1 — Supplementary figures. [file thnov09p6129s1.pdf]

Supplementary Figure 1

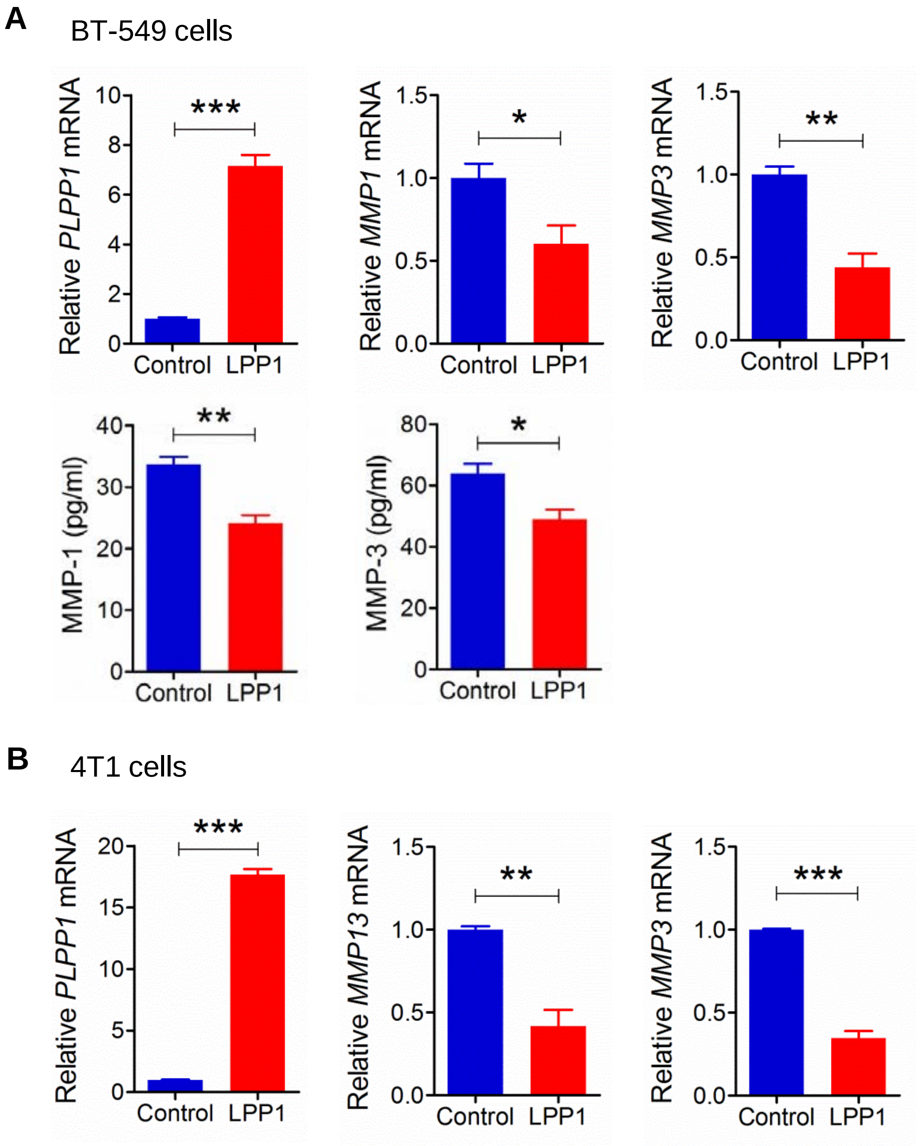

Supplementary Figure 1. **A:** Effects of increasing LPP1 (*PLPP1*) expression on mRNA and protein levels of MMP-1 and MMP-3 in BT-549 human breast cancer cells. **B:** Effects of increasing LPP1 (*PLPP1*) expression on mRNA levels of MMP-13 and MMP-3 in 4T1 mouse breast cancer cells. Results are means and SEM from three independent experiments and analyzed by Student's *t*-test. **P*<0.05, ***P*<0.01, ****P*<0.001.

Supplementary Figure 2

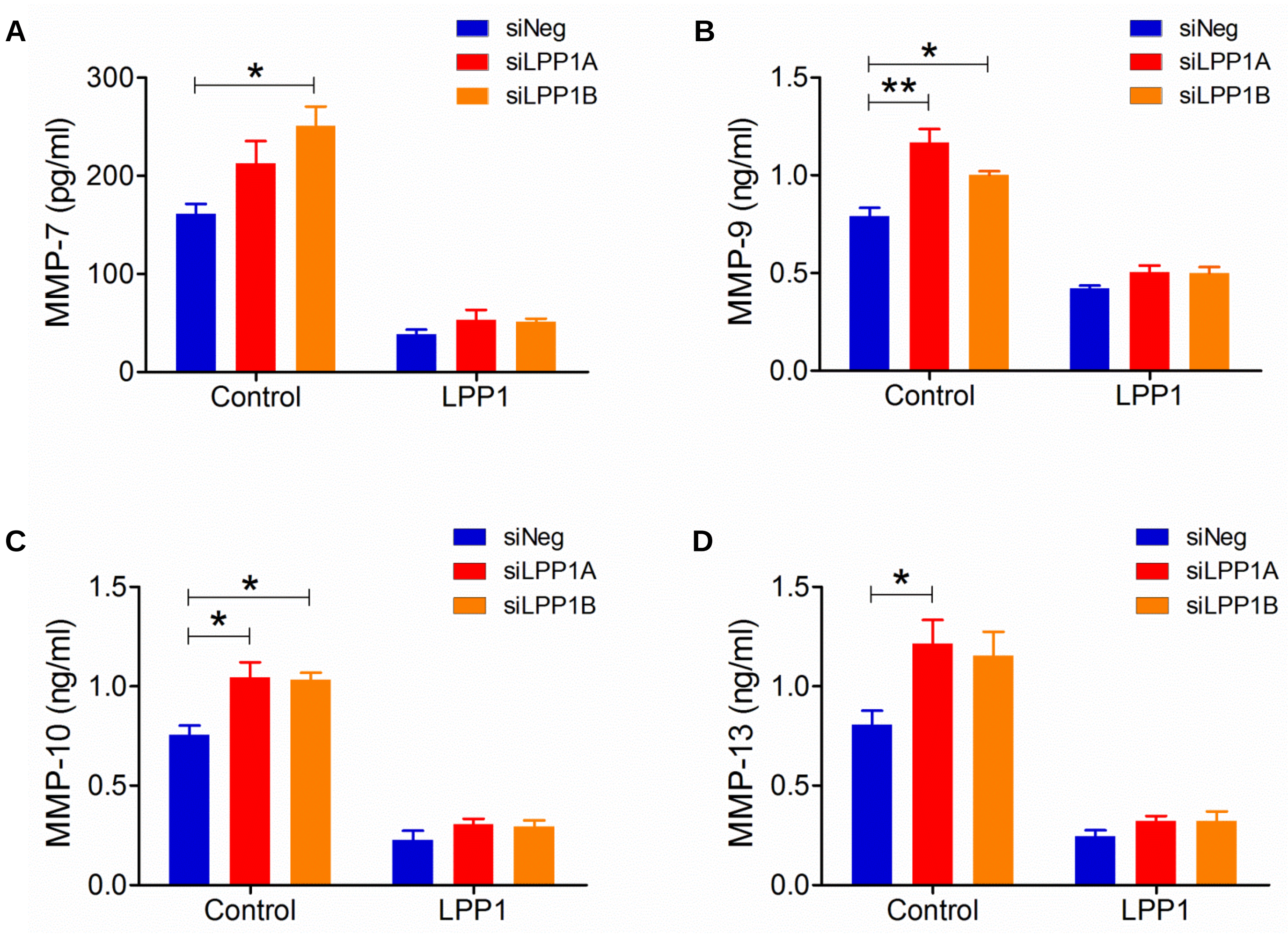

Supplementary Figure 2. Knockdown of LPP1 increased concentrations of MMP-7 (**A**), MMP-9 (**B**), MMP-10 (**C**) and MMP-13 (**D**) in the conditioned media from control MDA-MB-231 cells, but did not reverse the inhibition by LPP1 overexpression. Results are means and SEM and analyzed by ANOVA. n=6, *P<0.05, **P<0.01.

Supplementary Figure 3

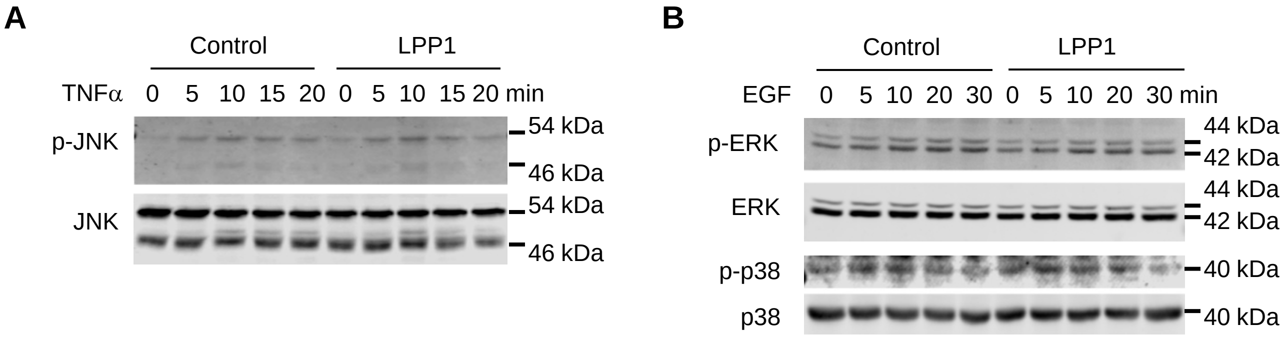

Supplementary Figure 3. **A:** Effects of TNFα (50ng/ml) on the JNK phosphorylation, and **B:** effects of EGF (100 ng/ml) on phosphorylation of ERK and p38 in MDA-MB-231 cells with or without increased expression of LPP1.

Supplementary Figure 4

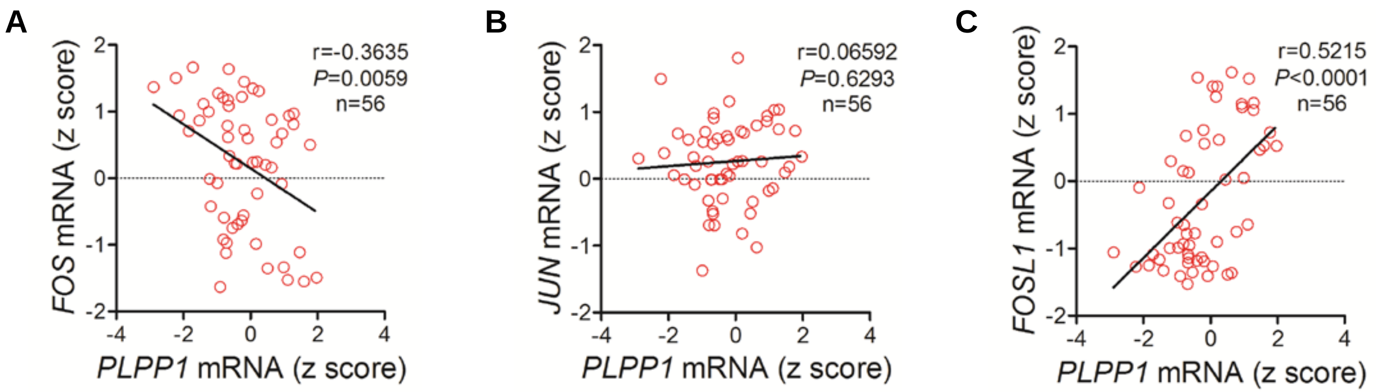

Supplementary Figure 4. **A:** Correlation between mRNA levels of LPP1 (*PLPP1*) and cFOS (*FOS*), and **B:** correlation between mRNA levels of LPP1 and cJUN (*JUN*), and **C:** correlation between mRNA levels of LPP1 and FRA1 (*FOSL1*) in 56 breast cancer cell lines. Data were obtained from Cancer Cell Line Encyclopedia of the Broad Institute and Novartis.

Supplementary Figure 5

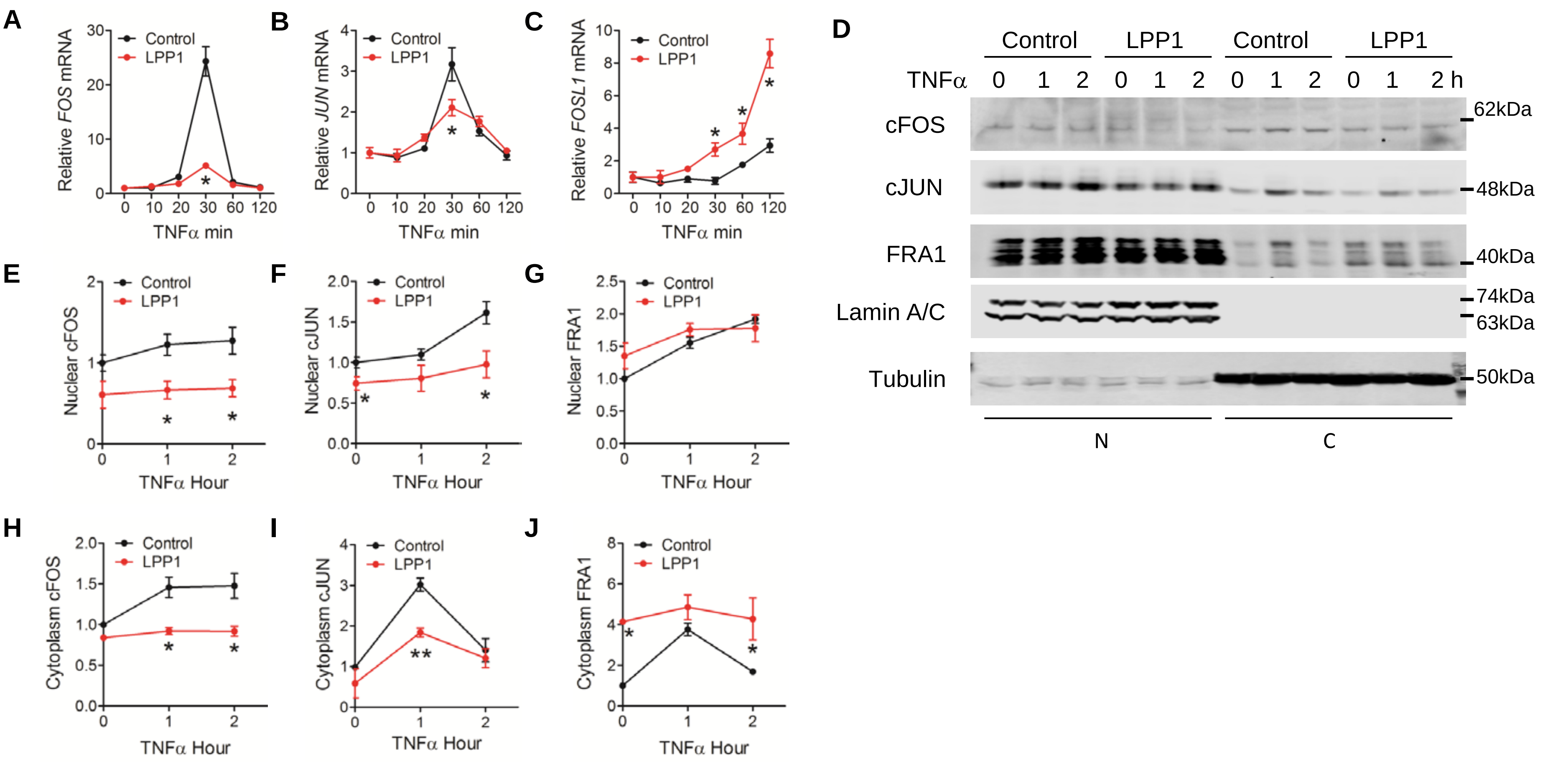

Supplementary Figure 5. **A, B** and **C:** Time course of cFOS (*FOS*), cJUN (*JUN*) and FRA1 (*FOSL1*) mRNA expressions induced by 50 ng/ml TNFα in MDA-MB-231 cell with or without increased LPP1. **D:** TNFα-induced expressions of cFOS, cJUN and FRA1 in cytoplasm (C) and nuclei (N) of MDA-MB-231 cells with or without increased LPP1. **E - J:** Quantification of the western blotting results. Results are means and SEM from three independent experiments, and analyzed by ANOVA. *$P < 0.05$, ** $P < 0.01$.

Supplementary Figure 6

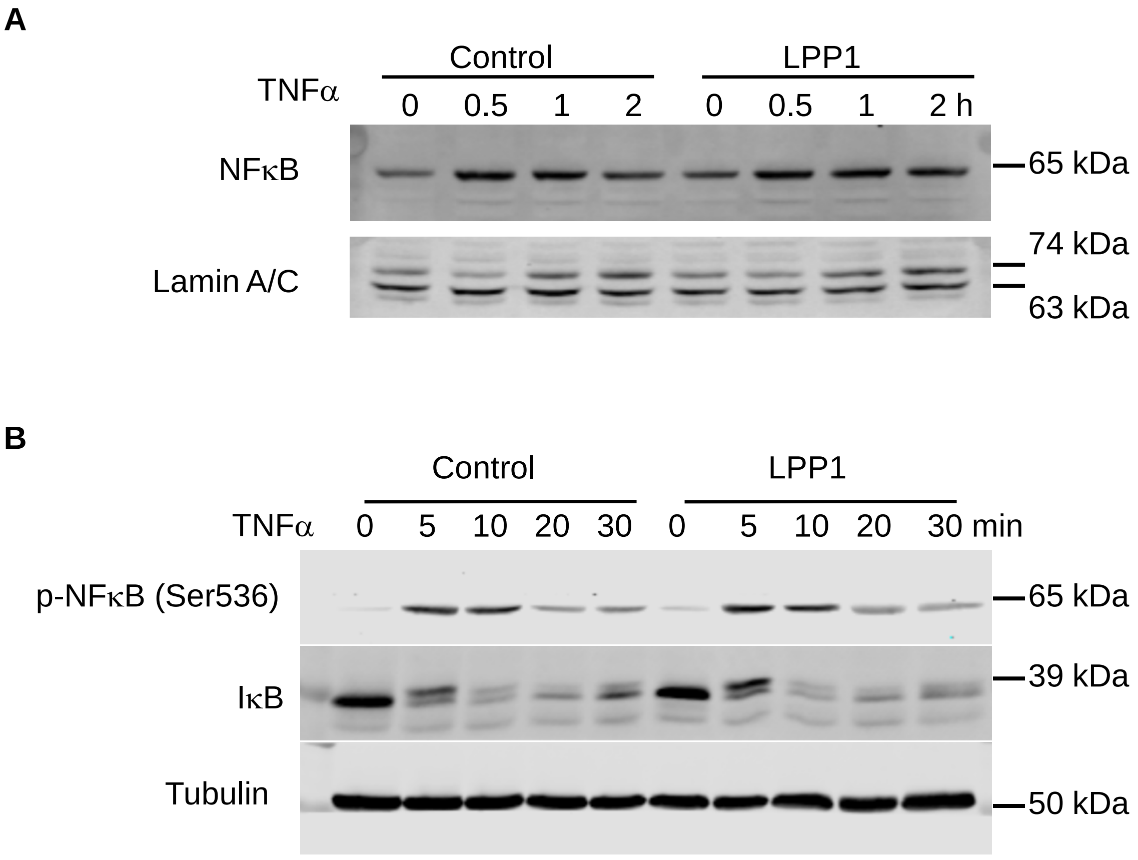

Supplementary Figure 6. **A:** TNFα (50 ng/ml) induced increase of NFκB in nuclei of control and LPP1 overexpressing MDA-MB-231 cells. **B**: TNFα (50 ng/ml) induced phosphorylation of NFκB and degradation of IκB in total cell lysates of control and LPP1 overexpressing MDA-MB-231 cells. Rabbit anti-NFκB (#8242), rabbit anti-phospho-NFκB (#3033) and mouse anti-IκB (#4814) antibodies were from Cell Signaling Technology.

Supplementary Figure 7

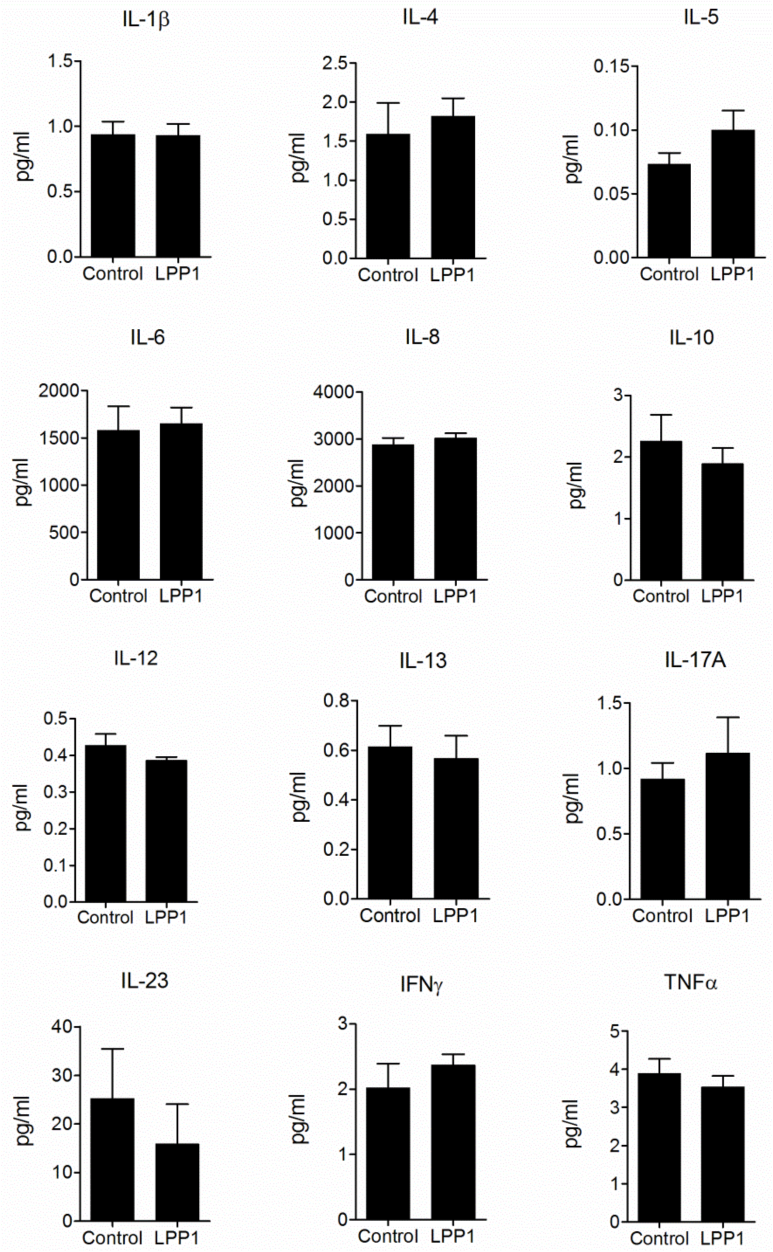

Supplementary Figure 7: Cytokine concentrations in the conditioned media of MDA-MB-231 cells with or without increased LPP1 expression. Results are means and SEM from three independent experiments.
